# MicroRNA Expression Profile Analysis of *Chlamydomonas reinhardtii* during Lipid Accumulation Process under Nitrogen Deprivation Stresses

**DOI:** 10.3390/bioengineering9010006

**Published:** 2021-12-27

**Authors:** Jingxian Zhang, Jiping Shi, Chenyang Yuan, Xiangcen Liu, Guilin Du, Ruimei Fan, Baoguo Zhang

**Affiliations:** 1 Lab of Biorefinery, Shanghai Advanced Research Institute, Chinese Academy of Sciences, No. 99 Haike Road, Pudong, Shanghai 201210, China; zhangjingxian@sari.ac.cn (J.Z.); shijp@sari.ac.cn (J.S.); yuanchy@shanghaitech.edu.cn (C.Y.); jpk9999@aliyun.com (X.L.); dugl1@shanghaitech.edu.cn (G.D.); 2 University of Chinese Academy of Sciences, Beijing 100049, China; 3 School of Life Science and Technology, ShanghaiTech University, Shanghai 201210, China; 4 Sino-UK Joint Laboratory for Brain Function and Injury and Department of Physiology and Neurobiology, Xinxiang Medical University, Xinxiang 453003, China

**Keywords:** microRNA, *Chlamydomonas reinhardtii*, lipid, nitrogen deprivation

## Abstract

Lipid accumulation in various microalgae has been found induced by nitrogen deprivation, and it controls many different genes expression. Yet, the underlying molecular mechanisms still remain largely unknown. MicroRNA (miRNAs) play a critical role in post-transcriptional gene regulation. In this study, miRNAs were hypothesized involved in lipid accumulation by nitrogen deprivation. A deep-sequencing platform was used to explore miRNAs-mediated responses induced by nitrogen deprivation in *Chlamydomonas reinhardtii*. The eukaryotic orthologous groups of proteins (KOG) function in the predicted target genes of miRNA with response to nitrogen deprivation were mainly involved in signal transduction mechanisms, including transcription, lipid transport, and metabolism. A total of 109 miRNA were predicted, including 79 known miRNA and 30 novel miRNA. A total of 29 miRNAs showed significantly differential expressions after nitrogen deprivation, and most of them were upregulated. A total of 10 miRNAs and their targeting genes might involve in lipid transport and metabolism biological process. This study first investigates nitrogen deprivation-regulated miRNAs in microalgae and broadens perspectives on miRNAs importance in microalgae lipid accumulation via nitrogen deprivation. This study provides theoretical guidance for the application of microalgae in bio-oil engineering production.

## 1. Introduction

The development toward clean and sustainable biofuels has gained significant support due to petroleum supply, global climate change, and energy shortage [1,2]. Compared with terrestrial biomass, microalgae have excellent photosynthetic efficiency, lipid content, and shorter cultivation time. Therefore, the search for a sustainable source of biofuel has renewed interest in microalgae as a potential feedstock [3]. Production of lipids by microalgae is usually because of environmental stresses, and many microalgae have the ability to accumulate large quantities of lipids when nutrient-deprived [4], particularly nitrogen deprived. For example, cyanobacteria showed an average increase of 9.8% in lipid content under nitrogen-depletion conditions [4]. The green microalga S. dimorphus (UTEX 1237) increased body lipids in N-limited cultures [5]. On average, an increase of 63%, 40%, and 23% in total lipids was obtained from Chlorella emersonii, Chlorella vulgaris, and Chlorella pyrenoidosa grown under nitrogen-depletion conditions, respectively [6]. In Chlorella vulgaris, the highest lipid content (ca. 36 wt.% on dry biomass) was obtained without nitrogen cultivation [7]. It has been reported that adding nitrogen and iron extracts to the nitrogen-deficient medium can promote the biomass accumulation of Chlorella and increase the lipid content [8]. Several recent studies have explored the mechanisms of lipids accumulation under nutrient deprivation. *Chlamydomonas reinhardtii*, as one of the most thoroughly characterized unicellular alga, has been mostly studied due to its rapid growth under nutritionally limited conditions and its low culture cost. The completion state of the Chlamydomonas genome, including sequence, assembly, and annotation, currently stands for a standard to determine if other algae should aspire [9]. Therefore, *Chlamydomonas reinhardtii* is undoubtedly the most suitable choice in solving the problems that this research focuses on. Many genes related to nitrogen import and assimilation have been found induced with nitrogen deprivation [10,11,12,13]. For example, in *Chlamydomonas reinhardtii*, the CrABCA2 protein level also increased through CrABCA2 expression upregulated under nitrogen deprivation conditions [14]. DGTT4 transcript was also upregulated notoriously under nitrogen depletion [12]. Nitrogen deprivation could activate a subset of control genes involved in gametogenesis, down-regulate protein biosynthesis, and lead to a marked redirection on its metabolism [15]. Recent studies have shown that down-regulating of the expression of flagella-associated WDR-containing protein CrFAP89 in *C. reinhardtii*, resulting in a decrease in fatty acid synthesis, but the transcription levels of CrFAP89 were significantly enhanced upon nitrogen deprivation [16]. Nitrogen deprivation also results in photosynthetic hydrogen production in *C. reinhardtii* [17]. However, its underlying molecular mechanisms about lipids accumulation under nitrogen deprivation are far from being completely understood, and the subcellular localization of the mechanism of TAG synthesis in microalgae is even unclear [18,19]. This work revealed changes in the expression of target genes by analyzing the major expression of microRNAs under nitrogen stress, and provided suggestions for the gene regulation of lipid accumulation in *Chlamydomonas reinhardtii* under nitrogen stress. This work fills in the knowledge gap of microRNAs regulation for gene expression under nitrogen stress.

MicroRNAs (miRNAs) are endogenous non-protein-coding RNAs with a length of approximately 22 nucleotides (nt), which is widely found in animals and plants, including unicellular green algae [20,21]. miRNAs could regulate the post-transcriptional gene expression by guiding both mRNA degradation and translational repression. Numerous studies have demonstrated that miRNAs play an important role in many biological and metabolic processes [22]. Identification of miRNAs expression profiles in organisms is a critical step to understanding the role of miRNAs executed in a specific biological process. Recently, more and more miRNAs profiles have been analyzed and detected out [23,24]. In addition, this discovery on large numbers of miRNAs has made some miRNAs find more important functions in the adaptive responses toward diverse abiotic stresses, including nutrient deficiency, cold, drought, high salinity, and UV-B radiation [20,25,26]. More interestingly, some miRNAs also have been found to play an important role in lipid metabolisms. For example, deletion of miR14 was found to be able to increase lipids accumulation in Drosophila [27]; miR-148a can regulate lipid metabolism by hepatic lipid content by inhibiting LDLR expression activity [28]. Compared with those in wild-type mice, ABCA1, ABCG1, and SREBP-1 on miR-33 related sterol metabolism were reduced in miR-33b knock-in (KI) mice, [29]; miR-122, triggered on key enzymes on the fatty acid synthesis and oxidation, also showed a relevant function for fatty acid metabolism. However, little information in microalgae under nitrogen deficiency stress is available at miRNA in the regulation of lipids accumulation.

Recently, high-throughput sequencing technology has been used as a powerful strategy to analyze miRNA expression patterns, detect novel miRNAs, and identify other functional small RNAs from an unprecedented perspective [30]. In this study, *Chlamydomonas reinhardtii*, which is usually the primary molecular model of microalgae, was subjected to deep sequencing and bioinformatics analysis. This work was performed to identify unknown and novel miRNAs and to determine major changes in miRNAs expression during nitrogen deprivation. These findings in this study would provide a novel insight into the molecular mechanisms that mediated nitrogen deprivation stress and a broader understanding of the induction of lipid accumulation in microalgae.

## 2. Materials and Methods

### 2.1. Strains and Culture Conditions

The *C. reinhardtii* CC-503 (cw92 mt+) used in this study were obtained from the Chlamydomonas Center, Duke University, Durham, NC, USA. The cells were grown in liquid cultures under continuous light (approximately 80 µmol photons m^−2^s^−1^). The culture medium used in this study included Tris-acetate phosphate (TAP) liquid culture and nitrogen-depleted TAP (TAP-N) culture medium [15]. TAP medium consisted of NH_4_Cl (7.48 mM), MgSO_4_ (406 µM), CaCl_2_ (340 µM), K_2_HPO_4_ (540 µM), KH_2_PO_4_ (463 µM), 20 mM Tris, 17.4 mM acetate, H_3_BO_3_ (184 µM), ZnSO_4_ (76.5 µM), MnCl_2_ (25.5 µM), FeSO_4_ (17.9 µM), CoCl_2_ (6.77 µM), (NH_4_)_6_Mo_7_O_24_ (0.88 µM), CuSO_4_ (6.29 µM) and Na_2_EDTA (148 µM). TAP-N medium was prepared from TAP by eliminating NH_4_Cl. For the nitrogen deprivation experiment, *C. reinhardtii* CC-503 was firstly grown in TAP culture medium to 5 × 10^6^ cells mL^−1^, followed by transfer to TAP-N culture medium for an additional 72 h. Cells cultures were grown in 500 mL shaker flasks with a culture volume of 100 mL with continuous shaking. Each experiment is repeated at least 3 times independently. The reagents used in this study were purchased from Shanghai Macklin Biochemical Co (Shanghai, China).

### 2.2. Lipid Analysis

#### 2.2.1. Nile Red Fluorescence Determination of Neutral Lipid

Nile red is a dibenzoxazine dye, easily soluble in organic solvents but extremely insoluble in water [31]. It is an excellent lysochrome dye and is widely used for dyeing lipid-producing microalgae [32]. Compared with the oil red o staining method, the Nile red staining method is easier to operate [31]. During the cultivation of *Chlamydomonas reinhardtii*, the lipophilic reagent Nile red is used to estimate the lipid content by colorimetry so that lipids can be directly quantified [8].

A stock solution of Nile red was prepared by adding 5 mg of Nile red to 100 mL of acetone [32]. The solution is stored in an amber bottle at 4 °C and protected from light. Transfer about 150 μL of algae cell suspension (TAP and TAP-N medium) to 96-well plates with a transparent bottom. Add 5 μL of Nile red solution to each well, mix well, and incubate for 15 min at 37 °C in the dark. Finally, a 96-well microplate reader (Synergy H1, Bio-Tek Instruments, Winooski, VT, USA) was used to quantify the cell neutral lipids with an excitation wavelength of 485 nm and an emission wavelength of 595 nm [33].

#### 2.2.2. Lipid Extraction

One hundred mg (dry weight) of algal cultures were harvested by centrifugation (5000× *g* × 5 min) and subsequently freeze-dried for at least 6 h. The lipids were extracted with 10 mL of chloroform:methanol (2:1), and the extract was centrifuged at 3000× *g* (Eppendorf centrifuge 5804R, Hamburg, Germany) for 10 min for phase separation [34]. Remove the upper layer (methanol water layer) and collect the lipid-containing chloroform layer. The chloroform was evaporated from the extract to produce the resulting algae oil, which was analyzed by GC-MS [33].

#### 2.2.3. Fatty Acid Determination

The fatty acid profile of the lipid sample was determined by converting the fatty acids in the lipid to fatty acid methyl esters (FAMEs). The FAME composition was determined using an Agilent 6890 N gas chromatography instrument coupled with an Agilent 5975 mass-selective detector and an Agilent autosampler 7683-B injector (Agilent Technologies, Palo Alto, CA, USA). A DB-624 capillary column with a dimension of 30 m × 0.32 mm ID × 1.80 μm film thicknesses (Agilent Technologies, Palo Alto, CA, USA) was used for the chromatographic separation. Details of the procedure have been described elsewhere [33]. Keep the column at 120 °C for 1 min, then increase it to 240 °C at a rate of 20 °C/min, and then keep it at 240 °C for 13 min. The transfer line between GC and MS was kept at 240 °C.

### 2.3. Small RNA Library Construction and Deep Sequencing

To generate material for high-throughput sequencing, *C. reinhardtii* CC-503 was grown in 100 mL of TAP culture medium to 5 × 10^6^ cells mL^−1^. The culture was divided into two halves, the cells were collected by centrifugation, and one pellet was resuspended in 50 mL TAP medium and the other pellet in 50 mL TAP-N medium. After 48 h, the total RNA was isolated from TAP and TAP-N cells as described above by using Trizol reagent (Invitrogen, Life Technologies, Carlsbad, CA, USA) according to the manufacturer’s instructions. The RNA quality and quantity were determined using an Agilent 2100 Bioanalyzer (Agilent, Santa Clara, California, USA).

The nitrogen deficiency small RNA library: equal quantities (20 μg) of total RNA isolated from TAP-N culture medium with three parallel experiments were mixed to construct.The control small RNA library: equal quantities (20 μg) of total RNA isolated from the TAP culture medium with three parallel experiments were mixed to construct. Then, the two different samples were subjected to 15% denaturing polyacrylamide gel electrophoresis, after which the small RNA fragments of 18–30 nt were isolated from the gel and purified. Next, the small RNA molecules have ligated a pair of Solexa adaptors to their 5′ and 3′ ends and then converted to DNA by RT-PCR. Finally, according to the manufacturer’s protocols (Beijing Genomics Institute, Beijing, China), approximately 20 mg of RT-PCR products were directly sequenced using So-lexa 1G Genome Analyzer.

### 2.4. Sequence Analysis

After Solexa sequencing, the adaptor/acceptor sequences were removed, the low-quality tags were filtered out, and the contamination of adaptor sequences was cleaned up. The resulting set of each unique sequence with associated read counts was referred to as clean sequence tags. For noncoding RNAs identification, the clean sequences were compared with rRNA, tRNA, snRNA, and snoRNA deposited in Rfam (http://www.sanger.ac.uk/Software/Rfam, accessed on 29 May 2021) and the GenBank noncoding RNA database (http://www.ncbi.nlm.nih.gov/, accessed on 29 May 2021). In addition, all sequences, discarding redundancy, were searched against miRBase (Release 14.0; http://microrna.sanger.ac.uk/, accessed on 29 May 2021) to identify known miRNAs in *C. reinhardtii*. Only the perfectly matched sequences were considered to be known miRNAs.

The prediction of the novel miRNA of *Chlamydomonas reinhardtii* is based on the previously established standards for plant miRNA prediction [35]. Sequences with a perfect match or with one mismatch were retained for further analysis. The secondary structures of the small RNA precursor were obtained using the RNAfold program (http://rna.tbi.univie.ac.at/cgi-bin/RNAWebSuite/RNAfold.cgi, accessed on 3 June 2021). Then, the novel miRNAs were identified using the MIREAP program developed by the BGI (Beijing Genome Institute, https://sourceforge.net/projects/mireap/, accessed on 5 June 2021). Finally, the RNA secondary structure was checked using Mfold.

### 2.5. The Nitrogen Deprivation-Responsive miRNAs in C. reinhardtii

To compare the miRNAs expression between *C. reinhardtii* cultured in TAP and TAP-N medium and to determine the responsive miRNAs in *C. reinhardtii* at nitrogen deprivation, the expression of miRNAs in two samples (culture with TAP and TAP-N medium, respectively) were normalized to obtain the expression of transcripts per million (TPM). If the normalized expression of a given miRNA is zero, its expression value will be modified to 0.01. If the normalized expression of a given miRNA is less than 1 in both samples, this miRNA is removed in future differential expression analysis [36].

The fold change between treatment and control was calculated as:

Normalized expression (NE) = Actual miRNAs sequencing count/Total count of clean reads ×1,000,000

Fold change = log_2_ (TAP-N/TAP)

Then, perform statistical analysis based on Poisson distribution. Calculate the *p*-value according to the formula [37].

$$P\left(x|y\right)=\left(\frac{N_{2}}{N_{1}}\right)\frac{\left(x+y\right)!}{x!y!{\left(1+\frac{N_{2}}{N_{1}}\right)}^{\left(x+y+1\right)}}\;$$

$$C=\left(\gamma \leq \gamma_{min}|x\right)=\overset{\gamma \leq \gamma_{min}}{\underset{\gamma =0}{\sum }}p(\gamma |x)\;$$

$$D=\left(\gamma \geq \gamma_{max}|x\right)=\overset{\infty }{\underset{\gamma \geq \gamma_{max}}{\sum }}p(\gamma |x)\;$$

The *N*_1_ and *x* represent the total count of clean reads and normalized expression level of a given miRNA in the small RNA library of the TAP library sample, respectively. The *N*_2_ and *y* represent the total count of clean reads and normalized expression level of a given miRNA in the small RNA library of the TAP-N library sample, respectively.

### 2.6. The Target Nitrogen Deprivation-Responsive miRNAs and KOGs Analysis

To understand the molecular function of the nitrogen deprivation-responsive miRNAs in *C. reinhardtii*, the target gene prediction was carried out as described by Allen et al. [35] and Schwab et al. [38]. The prediction was subject to the following rules: (1) between the small RNA and the target no more than four mismatches (G-U bases count as 0.5 mismatches); (2) no more than two adjacent mismatches in the miRNA: target duplex; (3) no adjacent mismatches in positions 2–12 of the miRNA: target duplex (5′ of miRNA); (4) no mismatches in positions 10–11 of the miRNA: target duplex; (5) No more than 2.5 mismatches in positions 1–12 of the miRNA: target duplex (5′ of miRNA); (6) The minimum free energy (MFE) of the miRNA/target duplex should be greater than 75% of the MFE of the miRNA bound to its perfect complement. The functional category of obtained target genes was annotated against the KOG database (http://genome.jgi-psf.org/cgi-bin/kogBrowser?db=Chlre4, accessed on 10 June 2021) using BLAST program with a cutoff of E value < 1 × 10^−5^.

## 3. Results

### 3.1. Analysis on Lipid and Fatty Acid Composition of C. reinhardtii under Nitrogen-Replete and Nitrogen-Deprived Conditions

**Figure 1:**
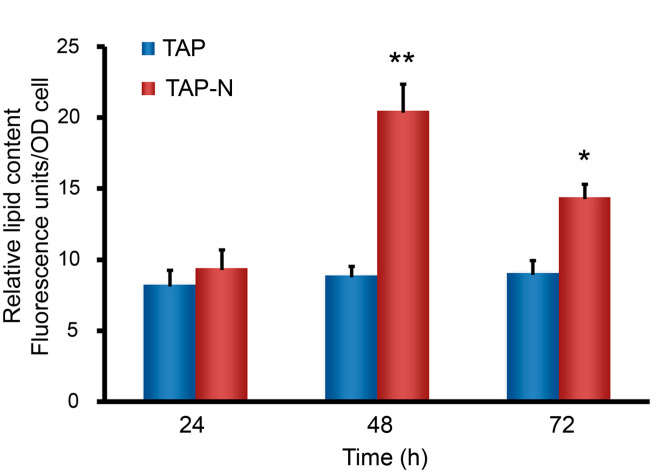
Lipid accumulation in nitrogen-deprived cells. *C. reinhardtii* cultures cultivated in nitrogen-replete medium (TAP) and nitrogen deficiency medium (TAP-N) were stained with Nile red on days 1, 2, and 3. Neutral lipid content was measured by determining Nile red fluorescence. * *p* < 0.05, ** *p* < 0.01 versus littermate controls by two-way ANOVA.

The Nile red fluorescence method, excellently applied to many microalgae, was used to determine the relative lipid content of *C. reinhardtii* under nitrogen-deprived conditions [32]. Cell density was determined by measuring the optical densities of the cultures. Figure 1 shows the relative neutral lipid content per OD unit under these culture conditions, as measured by Nile red fluorescence. After the two days of nitrogen deprivation, *C. reinhardtii* accumulated substantial amounts of lipid and but declined after day 3. However, cells in a nitrogen-replete medium accumulated relatively little lipid until day 3. This result showed that the lipid content of *C. reinhardtii* could be increased under nitrogen deprivation stress.

The chemical property of fatty acid esters, such as the unsaturation degree and carbon chain length, determine cetane number, viscosity, cold flow, oxidative stability, and iodine value related to biodiesel [39]. Therefore, the fatty acid profiles of lipid present in *C. reinhardtii* were analyzed by lipid extraction, transesterification, and fatty acid methyl esters (FAME) obtained by GC-MS analysis. The fatty acid profiles of the *C. reinhardtii* are presented in Table 1. Overall, C16:0, C18:0, C16:1ω, C18:2ω, C18:3ω, and C20:2ω were the major fatty acid under these two culture conditions. The most abundant saturated and unsaturated fatty acid was C16:0 (about 17–18%) and C18:3ω (about 24–32%), respectively. However, the total saturated fatty acids and the total unsaturated fatty acid displayed a significant interaction under nitrogen deficiency conditions. Under nitrogen deficiency conditions, *C. reinhardtii* contained a more saturated fatty acid (about 41.96%) but a lower unsaturated fatty acid (about 58.04%) as compared with that from normal conditions (about 34.26% and 65.74%, respectively).

### 3.2. Deep Sequencing of C. reinhardtii Small RNAs

**Figure 2:**
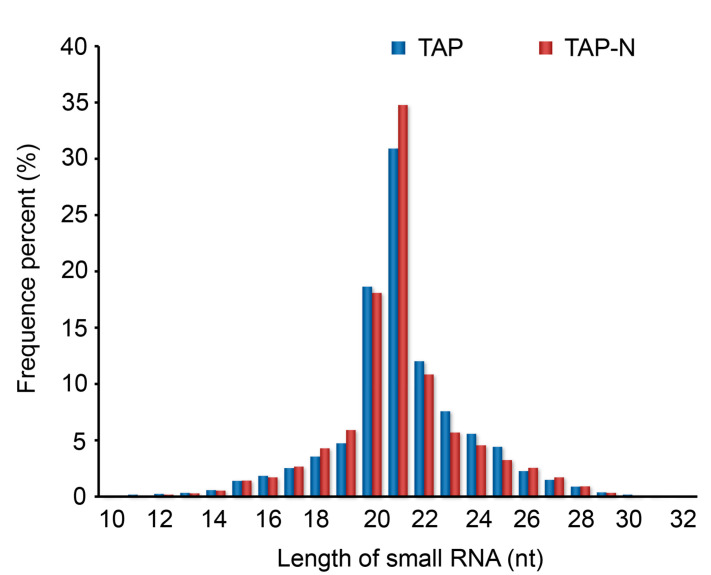
The length size distributions of small RNAs from the libraries of both TAP and TAP-N. The occurrences of each unique sequence read were counted to reflect relative expression level, and only small RNA sequences in the range of 10 to 32 nt were considered. TAP represents unique sequence reads of small RNAs of *C. reinhardtii* cultivated in nitrogen-replete medium; TAP-N represents unique sequence reads of small RNAs of *C. reinhardtii* cultivated in nitrogen deficiency medium.

To investigate *C. reinhardtii* small RNA under nitrogen deprivation stress, two small RNA libraries, TAP and TAP-N culture medium, were generated using the pooled RNA isolated from *C. reinhardtii*. These two *C. reinhardtii* small RNA libraries were sequenced by a deep-sequencing technology Solexa to produce highly accurate, reproducible, and quantitative readouts of small RNAs. Solexa sequencing of TAP and TAP-N libraries generated a total number of 15,360,483 and 16,105,446 raw reads, respectively. After removing adaptor/acceptor sequences, filtering out low-quality tags, and cleaning up the contamination from the adaptor-adaptor ligation, a total number of 28,362,008 clean reads from the two libraries (13,790,887 and 14,571,121, read from TAP and TAP-N libraries, respectively) were obtained with 891,186, and 1,403,685 unique reads, respectively. These unique sequences contained 220,552 common sequences between TAP and TAP-N libraries, 670,634 TAP-specific sequences, and 1,183,133 TAP-N-specific sequences (Table S1 and Figures S1 and S2), showing that TAP-N small RNA libraries had a higher unique sequences tag. The results also indicated that nitrogen deprivation affected the small RNA expression patterns in *C. reinhardtii*. Because small RNAs with known functions are commonly 20–24 nt long [40], the unique size distribution patterns of the small RNA sequence in the two libraries were also analyzed and showed that the majority of small RNA was 21 nt in size, with 20 nt, 22 nt, and 23 nt, which is consistent with the typical size of miRNAs from Dicer digestion products (Figure 2).

### 3.3. Identification and Profiling of Known miRNAs

**Figure 3:**
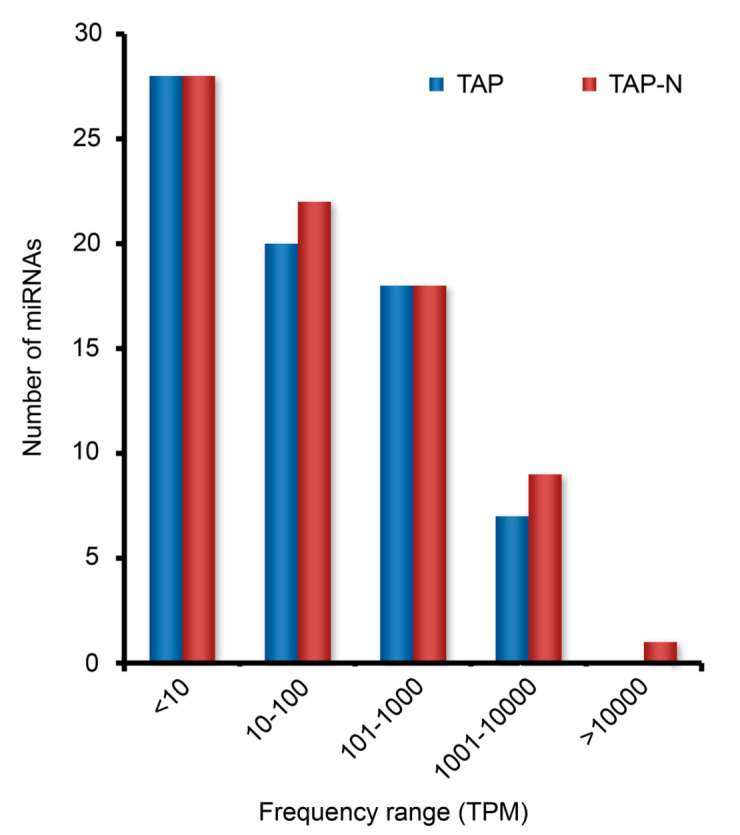
Frequency of read counts (TPM) for individual, known miRNAs presented in TAP and TAP-N libraries. The read count is based on the quantity of reads detected during the deep sequencing of the small RNA library for miRNAs detection was used the miRBase release 14.0. TAP represents read counts of individual, known miRNAs of *C. reinhardtii* cultivated in nitrogen-replete medium; TAP-N represents read counts of individual, known miRNAs of *C. reinhardtii* cultivated in nitrogen deficiency medium.

**Figure 4:**
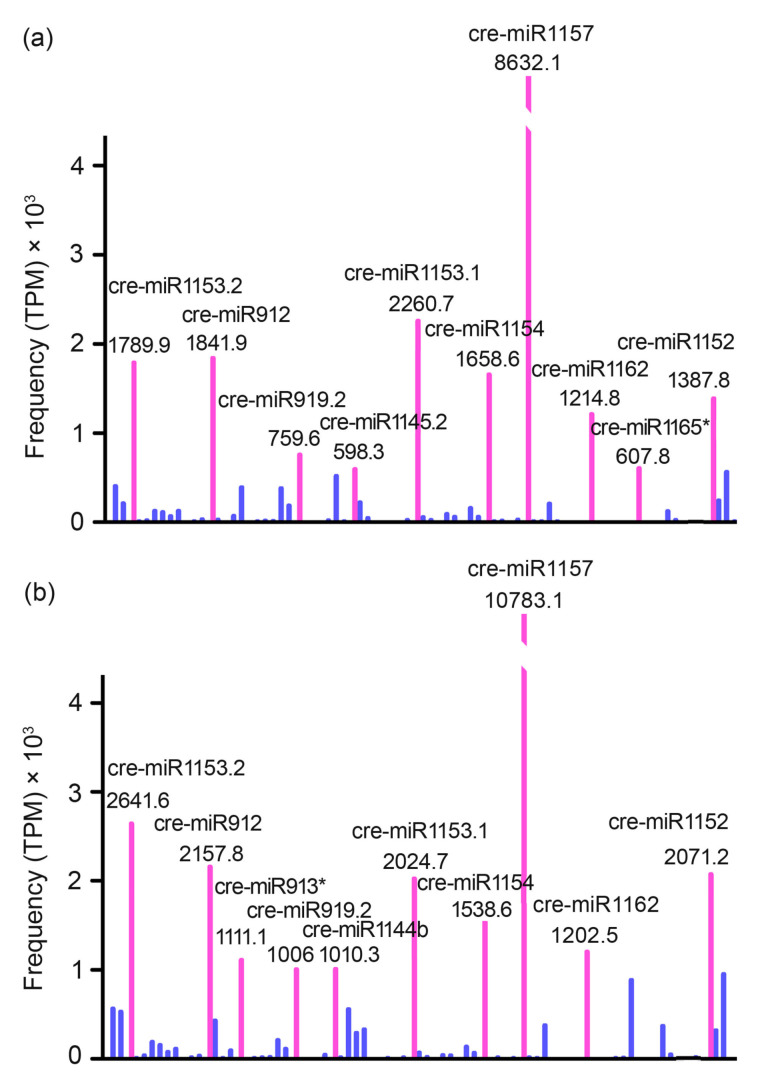
Abundance and differential expression of known miRNAs expressed (TPM) in *C. reinhardtii*. (**a**) represents the known miRNAs of *C. reinhardtii* cultivated in nitrogen-replete medium; (**b**) represents the known miRNAs of *C. reinhardtii* cultivated in nitrogen deficiency medium. Red lines indicated the TOP ten miRNAs; blue lines indicated the relatively less abundant miRNAs.

To identify known miRNA homologs in *Chlamydomonas reinhardtii*, annotate clean sequences based on their similarity with the mature miRNA sequences in miRBase (release 14.0). With this annotation, a total of 79 unique known miRNAs were identified, including 72 *C. reinhardtii* miRNAs overlapped between the two libraries of one miRNA detected only in the TAP library and 6 miRNAs detected only in the TAP-N library (Table S2). In a high-throughput sequencing, the relative expression level of a specific miRNA can be measured by the frequency of its transcript abundance, which could be counted by first normalizing the absolute sequence reads with transcripts per million (TPM) in the two libraries. Based on this measurement, the expression range of specific miRNAs was from less than 10 to more than 10,000 counts (Figure 3). Thus, the sequencing data revealed a wide range of expression levels spanning five times magnitude. For both libraries, the majority of miRNAs (>70%) were sequenced with a less than 10 TPM. cre-miR1157 was the most highly expressed miRNA in both libraries with 8632 TPM (~34% of all miRNAs reads) in the TAP library and 10,783 TPM (~33% of all miRNAs reads) in the TAP-N library (Figure 4, Table S2). Moreover, these two libraries shared eight out of the top 10 most frequently expression level miRNAs: cre-miR912, cre-miR919.2, cre-miR1152, cre-miR1153.1, cre-miR1153.2, cre-miR1154, cre-miR1157, and cre-miR1162 (Figure 4, Table S2). The sequencing frequency of some other miRNAs, including cre-miR1142, cre-miR1148.1, cre-miR1162*, and cre-miR1163.1, was low in both the libraries. These miRNAs may be expressed at a low level in certain cell types under a certain condition. To investigate the evolutionary roles of these known miRNAs, extensive comparisons have been made against known miRNAs in some other plant species and animals. The results showed no miRNAs with a direct sequence similarity between *C. reinhardtii* and higher plants/animals.

### 3.4. Potential Novel miRNAs

One most important features of deep sequencing are to be employed in the discovery of novel miRNAs. In this study, a high percentage of small RNAs, 66.61% in the TAP library and 58.75% in the TAP-N library were sorted as unannotated RNAs. To check whether novel miRNAs were present among unannotated RNAs. In this study, the miReap algorithm (http://sourceforge.net/projects/mireap, accessed on 5 June 2021) was employed to call all the candidate miRNAs precursors with hairpin-like structures perfectly mapped by sequencing reads (seen in material and methods). Subsequently, 30 putative novel miRNAs were identified from the two libraries (Table S3). Of these novel miRNAs, 23 miRNAs were detected in both TAP and TAP-N samples, 5 miRNAs were identified only in the TAP sample, and 2 miRNAs were identified only in the TAP-N sample (Table S3).

The size range of these newly identified miRNAs sequences was from 20 to 23 nt, and the length of their predicted hairpin structures was from 62 to 96 nt, similar to known *C. reinhardtii* miRNAs. A total of 30 putative novel miRNAs had a negative folding free energy ranging from −54.3 to −22.3 kcal mol^−1^ (average value = −32.4 kcal mol^−1^) according to Mfold. Among these 30 novel miRNAs, 21 miRNAs were encoded by a single copy in the *C. reinhardtii* genome, whereas the other 9 miRNAs had multiple loci (Table S3), probably leading to duplication events that were still active in the *C. reinhardtii* genome. For these 9 miRNAs, most of them only had 2–5 loci, and 1 miRNA had 9 loci in the genome.

To understand the conservation of the new identified *C. reinhardtii* miRNAs, the novel miRNAs were compared to genomes of species by representing important lineages. The results show that all the novel miRNAs do not have a sequence homologous with the miRNAs gene of other species, which indicated species-specific miRNAs identified in *C. reinhardtii* consistent with the evolutionary character of the known miRNAs in *C. reinhardtii*.

### 3.5. Response of C. reinhardtii miRNAs to Nitrogen Deprivation Stress

The deep-sequencing approach can be used as a powerful tool for profiling miRNAs expression. The change in miRNAs frequency between the nitrogen deprivation (TAP-N) and control (TAP) libraries might indicate that nitrogen deficiency stress regulates their expression. To determine the response of *C. reinhardtii* miRNAs for nitrogen deprivation, the TPM value changes were compared between TAP-N and TAP libraries to find the miRNAs that were up-or down-regulated under nitrogen deficiency stress in *C. reinhardtii*. Meantime, to minimize noise and improve accuracy, miRNAs with a frequency no less than 1 TPM value in at least one library were selected for comparison.

**Figure 5:**
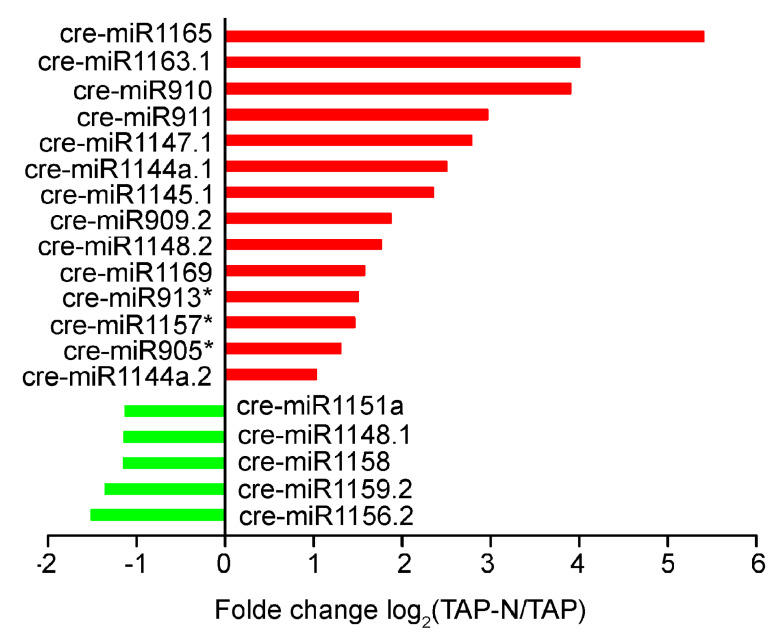
Differentially expressed known miRNAs in response to nitrogen deficiency in *C. reinhardtii*. The significantly differentially expressed miRNAs with greater than 2-fold relative change and *p*-value < 0.05 are shown. * *p* < 0.05.

Overall, approximately half of known miRNAs with greater than 1.5 fold relative change in TPM value were identified between the two libraries (Table S2). After the nitrogen deprivation stress, 19 known miRNAs showed an obvious change (fold-change > 2 and *p*-value < 0.05) (Figure 5, Table S2). Of the 19 known miRNAs, 14 miRNAs were upregulated, and 5 miRNAs were down-regulated. The most obvious change was observed for cre-miR1165, whose expression level increased about 45-folds in the TAP-N library compared with that of the TAP library. The expression of cre-miR910, cre-miR911, cre-miR1147.1, cre-miR1145.1, and cre-miR1163.1 also showed more than 5-folds increasing in the TAP-N library.

In addition, comparing the standardized sequence reads of the new miRNAs between the two libraries showed that the 10 new miRNA sequences also have significant changes (fold-change > 2 and *p*-value < 0.05), so there may be differential expression (Table 2, Table S3). Among the 10 predicted novel miRNAs with adjusted expression under nitrogen deprivation stress, 8 miRNAs were upregulated, and 2 miRNAs were down-regulated. A total of 2 novel miRNAs (cre-miR-new24 and cre-miR-new25) were only expressed in the TAP library, while 5 novel miRNAs (cre-miR-new26, cre-miR-new27, cre-miR-new28, cre-miR-new29, and cre-miR-new30) were only expressed in the TAP-N library. Analysis of the isolation frequencies for these 29 nitrogen deficiency stress-responsive miRNAs, including 19 conserved miRNAs and 10 novel miRNAs, indicated that most of these genes belonged to the less expressed miRNAs, with the isolation frequencies less than 100 TPM.

### 3.6. The Target of Nitrogen Deficiency-Responsive miRNAs and KOGs Analysis

To understand the 19 conserved miRNAs and 10 novel miRNAs biological function for the nitrogen deficiency responsive (Figure 4 and Table 2), potential *C. reinhardtii* targets using the Filtered Gene Set transcripts (Release v4.0) of the *C. reinhardtii* genome sequence (http://genome.jgi-psf.org/Chlre4/Chlre4.download.ftp.html, accessed on 12 June 2021) was computationally predicted. The computational pipeline predicted 204 unique putative genes that were targeted by 29 nitrogen deficiency-responsive miRNAs sequences (Table S4).

The putative target genes subjected to the eukaryotic orthologous groups of proteins (KOG) analysis identified the biological processes actively regulated by the nitrogen deficiency-responsive miRNAs in *C. reinhardtii*. The targets were annotated by using the KOG annotations available from the Chlre4_KOG (http://genome.jgi-psf.org/Chlre4/Chlre4.download.ftp.html, accessed on 12 June 2021).

**Figure 6:**
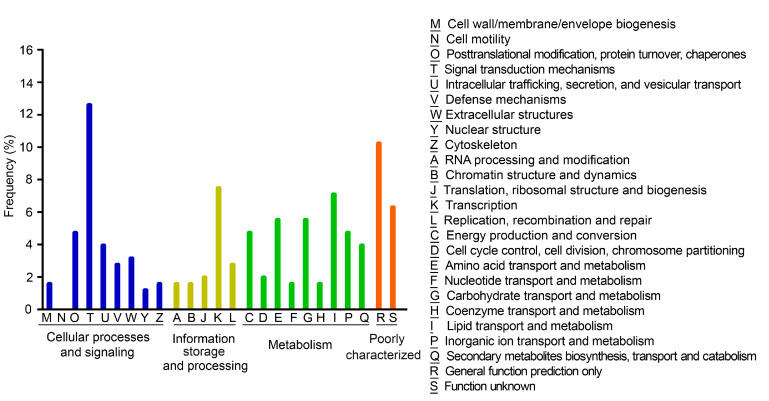
Functional assignments to the KOG categories. The graph shows the assignment of the targets of the nitrogen deficiency-responsive miRNAs in *C. reinhardtii* to the 25 categories of the eukaryotic orthologous groups of proteins (KOG). The main KOG categories are represented in different colors. The nitrogen deficiency-responsive miRNAs included 19 known miRNAs and 10 novel miRNAs.

The putative target genes were found involved in a wide variety of biological processes. These were the four uniquely assigned main KOG classes (31.50% for “Cellular Processes and Signaling”, 15.35% for “Information Storage and Processing”, 36.61% for “Metabolism”, 16.54% for “Poorly characterized”). The main represented sub-categories were “Signal transduction mechanisms” (12.60%) in the “Cellular Processes and Signaling” main class, “General function prediction only” (10.24%) in “Poorly Characterized” main class, “Transcription” (7.48%) in “Information Storage and Processing” main class and “Lipid transport and metabolism” (7.09%) of the “Metabolism” main class (Figure 6).

Particularly, 13 targets genes of the nitrogen deficiency-responsive miRNAs attend lipid transport and metabolism pathways (Table 3). This result indicated that some nitrogen deficiency-responsive miRNAs might play a role in lipid metabolism cascades through regulating the activity of their target genes. To understand the role of miRNAs in lipid metabolism cascades, we further performed a deep-sequencing approach to determine the expression patterns of putative 13 target genes that are involved in lipid transport and metabolism pathways, which investigate the correlation of miRNAs and these predicted targets [41]. After miRNAs analysis, we determined the fold changes of the target genes in *C. reinhardtii* under nitrogen deficiency stress. Apart from a few exceptions, 11 of 13 miRNAs-target gene pairs studied in *C. reinhardtii* showed opposite expression patterns in response to nitrogen deficiency stress, which is consistent with miRNAs function in guiding the cleavage of target mRNAs. However, positive correlations were also observed between cre-miR910/cre-miR1163 and their putative target acyl-CoA oxidase/START domain-containing proteins, respectively. The positive correlation indicates that the miRNAs were coexpressed with their targets, which speculate miRNAs related to suppress the encoded protein translation of their targets rather than by leading mRNA cleavage [42].

## 4. Discussion

Recently, the investigation of algae miRNAs has gained more attention due to their vital function in metabolic processes and adaptation to various stresses. Currently, different algae miRNAs genes, including *Chlamydomonas reinhardtii* [43,44,45], *Chlorella sorokiniana* [46], and *Botryococcus braunii* [47], have been identified, and some of them also have been well characterized. However, the role of miRNAs in lipid accumulation under nitrogen deprivation stress for microalgae is still unclear.

In this work, a total number of 109 miRNAs were detected in the growth of *C.reinhardtii* in both nitrogen-replete (TAP + N) and nitrogen-depleted (TAP-N) conditions. *C. reinhardtii* miRNAs identified in the present study provided more information in the miRNAs profiling. The Solexa deep-sequencing approach used in this work may be more sensitive enough to detect more miRNAs expressed at a very low level in *C. reinhardtii.* Besides these known miRNAs, 30 novel miRNAs were found out by Solexa deep sequencing, which increased the number of accounts of *C. reinhardtii* miRNAs families. The 109 miRNAs were compared with genomes of species representing important lineages for investigating their evolutionary roles in other organisms. No homolog of these *C. reinhardtii* miRNAs was found in other organisms, suggesting that these miRNAs are *C. reinhardtii*-specific miRNAs, and there *C. reinhardtii* miRNAs may be evolved in an independent way, which coincided with the previous reports [48]. Different from higher eukaryotes, most miRNAs are high evolutionarily conserved in related species. These nonconserved *C. reinhardtii* miRNAs may play a more species-specific role [49].

Although some miRNAs are stress-regulated and involved in plant responses to nutrient deprivation [25], little information is available for microalgae in this area. This study on the nitrogen deprivation-responsive miRNAs in *C. reinhardtii* will provide a piece of useful information for understanding the molecular mechanism of lipid accumulation under nitrogen deficiency stress for microalgae. Similar to the previous research in plants [50], the results in this study showed that most of miRNAs expression in *C. reinhardtii* were affected by nitrogen deficiency stress, which indicated that the nitrogen response process is complex and requires a large number of miRNAs to participate in transcriptional and post-transcriptional gene expression regulation [51]. Interestingly, several reported sulfur deprivation-responsive miRNAs in *C. reinhardtii* are also involved in nitrogen deprivation stress. For example, cre-miR910, cre-miR1144a.1, cre-miR1147, and cre-miR1148.2 were significantly induced by sulfur deprivation in *C. reinhardtii* [51]. In this study, cre-miR910, cre-miR1144a.1, cre-miR1147, and cre-miR1148.2 were significantly induced under nitrogen deprivation stress in *C. reinhardtii*, which indicated these miRNAs may control one of the most critical defense systems for *C. reinhardtii* nutrition deprivation stress.

To better understand the biologic functional implication for the nitrogen deficiency-responsive miRNAs in *C. reinhardtii*, their targeted mRNAs and function enrichment were investigated. As a result, a total number of 204 potential targeted genes were identified with the rules of target prediction reported by Allen et al. [35]. To evaluate the functional enrichment, these predicted miRNA targets with KOG term annotation were annotated in this study. Predicted miRNA targets populated many KOG categories. Inconsistent with previous studies [15], the most significant KOG terms were genes involved in a signal transduction mechanism, including signal transduction factor casein/tyrosine kinase, actin regulatory protein, sensory transduction histidine kinase, cyclin-dependent kinase inhibitor, and growth factor receptor-bound proteins, indicating the important role of nitrogen deficiency-responsive miRNAs in both signal transduction and post-transcriptional regulation. Furthermore, KOG terms associated with some *C. reinhardtii* transcription factors such as TFIID, SMRT/SMRTER, DEAD-box superfamily, and TAZ Zn-finger proteins, indicating the post-transcription regulation function of nitrogen deficiency-responsive miRNAs.

It is noteworthy that some lipid transport and metabolism biological processes were also enriched in lipid metabolic regulation. The functional annotation chart of target genes for the nitrogen deficiency-responsive miRNAs revealed some factors involved in the regulation of lipids biosynthesis (acyl-CoA synthetase, fatty acid desaturase), lipid transport (long-chain acyl-CoA transporter), and fatty acid beta-oxidation (Pristanoyl-CoA/acyl-CoA oxidase), were all predicted, indicating that various aspects in the lipid metabolic process may be regulated by some nitrogen deficiency-responsive miRNAs in *C. reinhardtii*. A recent study found that under nitrogen stress, *C. reinhardtii* lipids synthesize related proteins: acetyl CoA synthetase, two fatty acid desaturases, and a phospholipase-B-like protein change rapidly, indicating a trend of transformation from metabolism to TAG synthesis at the post-transcriptional level [52], which is consistent with this study. Similarly, LIP4, a putative TAG lipase in *C. reinhardtii,* is transcriptionally upregulated in the presence of nitrogen deficiency and TAG accumulation [53]. Acyl-CoA oxidase is the target of cre-miR910, and it is the first enzyme in the fatty acid beta-oxidation pathway, which catalyzes the desaturation of acyl-CoAs to 2-trans-enoyl-CoAs. cre-miR1169 targets lysophospholipase in the regulation of glycerophospholipid metabolism. cre-miR1163.1 targets fatty acid desaturase that catalyzes the biosynthesis of highly unsaturated fatty acids (HUFA) from precursor essential fatty acids. cre-miR1144a.2 targets animal-type fatty acid synthase and related proteins. The protein belongs to the beta-ketoacyl-ACP synthases family that catalyzes the condensation of malonyl-ACP with the growing fatty acid chain. cre-miR-new16 targets a long-chain acyl-CoA transporter that catalyzes the pre-step reaction for β-oxidation of fatty acids or can be incorporated in phospholipids. cre-miR-new19 targets acyl-CoA synthetase that catalyzes the conversion of acetate into acetyl CoA as a precursor for fatty acid synthesis. These results may indicate that the differential expression profile of *C. reinhardtii* miRNAs led to massive changes in gene expression and lipid metabolism closely associated with triacylglycerol (TAG) accumulation of *C. reinhardtii*. Further experiments are needed to verify this hypothesis.

Based on the deep-sequencing technology, 79 knowns and 30 novel miRNAs were discovered from *C. reinhardtii* cells under nitrogen deprivation stress. In addition, deep-sequencing technology can be used as a powerful miRNAs expression profiling tool to identify the differentially expressed miRNAs, providing a basis for future miRNAs functions research and elucidating the potential mechanisms of regulating diverse molecular and physiological pathways. In this study, a total number of 29 miRNAs show differential responses to nitrogen deficiency stress in *C. reinhardtii*.

Target predictions revealed that lipid metabolic processes may be affected by changing miRNAs expression, which opens miRNAs tantalizing possibility involved in the lipid biosynthesis cascades. This would have a significant impact on the recent approaches that use miRNAs to increase the accumulation of lipid in microalgae.

## 5. Conclusions

This study proves that under nitrogen stress, the miRNA in *Chlamydomonas reinhardtii* is differentially expressed, most of which are upregulated. The putative miRNA target gene is predicted by KOG. It is predicted for the first time that 10 miRNAs and their target genes may be involved in lipid transport and metabolic biological processes.

This study provides suggestions on gene regulation to further explain the molecular mechanism of lipid accumulation in *Chlamydomonas reinhardtii* under nitrogen stress.

As energy pressure continues to increase, the use of biomass oils has received more attention. Microalgae has become an excellent cell factory for obtaining bio-oil due to its low cultivation cost. The use of bioengineering methods to improve the accumulation of microalgae oil and find a balance between microalgae biomass accumulation and oil production is the direction of our next efforts.

## Figures and Tables

**Table 1:** Fatty acid profile (percentage of total fatty acids) of extracted lipids from *C. reinhardtii,* which were cultivated in nitrogen-replete medium (TAP) and in nitrogen deficiency medium (TAP-N). Values are means of three replicates. Cells harvested after 48 h of cultivation.

Fatty Acid (%)	*C. reinhardtii* Based Biodiesel	
	TAP	TAP-N
C14:0	1.40 ± 0.43	3.95 ± 0.13
C16:0	17.05 ± 0.28	18.07 ± 0.70
C16:1ω7	1.47 ± 0.39	3.00 ± 0.24
C18:0	12.17 ± 0.46	11.75 ± 0.69
C16:1ω9	8.27 ± 0.91	10.59 ± 0.14
C18:2ω6	4.49 ± 0.94	7.87 ± 1.35
C20:0	1.56 ± 0.48	3.52 ± 0.59
C18:3ω3	32.63 ± 1.79	24.40 ± 0.63
C21:0	1.25 ± 0.34	2.19 ± 0.38
C20:2ω6	13.45 ± 0.72	9.22 ± 0.87
C20:3ω6	2.19 ± 0.11	2.24 ± 0.11
C22:4ω6	0.94 ± 0.43	0.33 ± 0.43
Other	3.13 ± 1.14	2.88 ± 0.25
∑Unsaturat	65.74 ± 2.58	58.04 ± 1.88
∑Saturat	34.26 ± 1.23	41.96 ± 1.52

**Table 2:** Expression changes of predict novel miRNAs in response to nitrogen deprivation stress in *C. reinhardtii*. TAP represents normalized expression level of novel miRNAs of *C. reinhardtii* cultivated in nitrogen-replete medium; TAP-N represents normalized expression level of novel miRNAs of *C. reinhardtii* cultivated in nitrogen-depleted medium; * fold-change (log_2_) > 1 or fold-change (log_2_) < −1, and 0.01 < *p*-value < 0.05; ** fold-change (log_2_) > 1 or fold-change (log_2_) < −1, and *p*-value < 0.01; -^1^ specifically expressed in TAP-N library; +^2^ specifically expressed in TAP library.

Novel miRNA Name	Normalized Expressio (TPM)		Fold-Change (log_2_(TAP-N/TAP))	*p*-Value	Sig-Lable
	TAP	TAP-N			
cre-miR-new1	0.65	1.17	0.8381	0.163	
cre-miR-new2	64.68	33.15	−0.9644	7.98 × 10^−34^	
cre-miR-new3	34.08	22.85	−0.5765	1.89 × 10^−8^	
cre-miR-new4	129.80	88.87	−0.5464	1.38 × 10^−25^	
cre-miR-new5	2.47	16.33	2.7280	2.96 × 10^−13^	**
cre-miR-new6	1.23	1.51	0.2926	0.542	
cre-miR-new7	95.14	123.33	0.3744	0	
cre-miR-new8	0.87	4.60	2.4017	3.45 × 10^−7^	**
cre-miR-new9	0.65	1.44	1.1430	0.046	*
cre-miR-new10	0.87	1.10	0.3356	0.553	
cre-miR-new11	88.39	139.45	0.6578	1.41 × 10^−13^	
cre-miR-new12	1.89	3.23	0.7748	0.026	
cre-miR-new13	7.90	7.89	−0.0021	0.989	
cre-miR-new14	33.28	9.95	−1.7418	5.51 × 10^−43^	**
cre-miR-new15	179.25	170.61	−0.0712	0.08	
cre-miR-new16	3.55	7.76	1.1261	2.23 × 10^−6^	**
cre-miR-new17	0.65	3.02	2.2101	4.22 × 10^−6^	**
cre-miR-new18	8.77	20.11	1.1965	3.55 × 10^−14^	**
cre-miR-new19	365.68	132.11	−1.4688	0	**
cre-miR-new20	5.80	73.84	3.6701	0	**
cre-miR-new21	18.20	23.95	0.3961	8.60 × 10^−4^	
cre-miR-new22	72.66	142.06	0.9674	2.63 × 10^−13^	
cre-miR-new23	24.58	50.79	1.0468	1.79 × 10^−13^	**
cre-miR-new24	0.00	4.25	-^1^	2.22 × 10^−16^	
cre-miR-new25	0.00	12.63	-^1^	2.22 × 10^−16^	
cre-miR-new26	7.18	0.00	+^2^	9.70 × 10^−32^	
cre-miR-new27	5.44	0.00	+^2^	3.18 × 10^−24^	
cre-miR-new28	17.98	0.00	+^2^	2.13 × 10^−78^	
cre-miR-new29	29.80	0.00	+^2^	1.93 × 10^−129^	
cre-miR-new30	44.88	0.00	+^2^	1.42 × 10^−194^	

**Table 3:** Validation of the predicted genes involved in lipid transport and metabolism targeted by nitrogen deficiency-responsive miRNAs through high-throughput sequencing.

miRNA Expression			Target Gene Expression			
miRNA	Fold Change	Expression	Gene Product	Fold Change	Expression	*p*-Value
cre-miR1156.2	0.35	Down	Prosaposin	2.17	Up	0
cre-miR1144a.2	2.04	Up	SAM-dependent methyltransferases	0.07	Down	2.12 × 10^−38^
			Animal-type fatty acid synthase and related proteins	0.24	Down	0.302
cre-miR1157*	1.25	Up	Cytochrome P450 CYP4/CYP19/CYP26 subfamilies	0.76	Down	0.284
cre-miR1163.1	16.71	Up	Fatty acid desaturase	0.28	Down	4.921 × 10^−2^
			Sterol C5 desaturase	0.86	Down	0.611
			START domain-containing proteins	1.25	Up	1.55 × 10^−3^
cre-miR1169	2.98	Up	Lysophospholipase	0.60	Down	0.135
cre-miR910	15.55	Up	acyl-CoA oxidase	1.34	Up	4.70 × 10^−2^
cre-miR-new5	6.61	Up	Putative phosphoinositide phosphatase	0.35	Down	0.146
cre-miR-new14	0.30	Down	3-Methylcrotonyl-CoA carboxylase	3.66	Up	0
cre-miR-new16	2.19	Up	Long-chain acyl-CoA transporter	0.46	Down	4.66 × 10^−3^
cre-miR-new19	0.36	Down	Acyl-CoA synthetase	4.13	Up	3.24 × 10^−12^

## Data Availability

The data provided in this study can be obtained in the figures and tables in this article and the figures and tables in the supplementary materials.

## Supplementary Materials

The following supporting information can be downloaded at: https://www.mdpi.com/article/10.3390/bioengineering9010006/s1, Figure S1: Ven chart for unique small RNAs; Figure S2: Ven chart for total small RNAs; Table S1: Summary of the common and specific sequences (≥18 nt) between TAP-N and TAP libraries; Table S2: Abundance and differential expression of known miRNAs expressed both in TAP and TAP-N samples; Table S3: predict novel miRNA in our small RNA libraries; Table S4: Complete list of predicted miRNA targets of nitrogen deficiency-responsive miRNA and KOGs annotations.
