# Supplementary material for: MicroRNA Expression Profile Analysis of Chlamydomonas reinhardtii during Lipid Accumulation Process under Nitrogen Deprivation Stresses
Source: Bioengineering (Basel). 2021 Dec 27;9(1):6. doi: 10.3390/bioengineering9010006 (PMC8773410; doi:10.3390/bioengineering9010006)
Supplement: Supplementary file 1 [file bioengineering-09-00006-s001.zip › Additional file 2.pdf]

Additional file 2. Abundance and differential expression of known miRNAs expressed both in TAP and TAP-N samples.

Table S2 Abundance and differential expression of known miRNAs expressed both in TAP and TAP-N samples.

miRNA name	Normalized expressio (TPM)		Fold-change[$\log_2$ (TAP-N / TAP)]	P-value	Sig-lable
	TAP	TAP-N			
cre-miR1157	8632.15	10783.11	0.3210	0	
cre-miR1153.1	2260.70	2024.69	-0.1591	5.40E-42	
cre-miR912	1841.94	2157.83	0.2284	6.91E-79	
cre-miR1153.2	1789.88	2641.59	0.5615	0	
cre-miR1154	1658.63	1538.59	-0.1084	1.31E-15	
cre-miR1152	1387.80	2071.15	0.5776	0	
cre-miR1162	1214.79	1202.52	-0.0146	0.35	
cre-miR919.2	759.63	1006.03	0.4053	3.55E-108	
cre-miR1165*	607.79	884.97	0.5420	2.92E-161	
cre-miR1145.2	598.29	558.09	-0.1004	8.52E-06	
cre-miR1172.2	566.61	952.36	0.7492	1.94E-306	
cre-miR1144b	519.18	1010.29	0.9604	0	
cre-miR905	407.23	566.39	0.4760	2.08E-82	
cre-miR913*	392.14	1111.10	1.5025	0	**
cre-miR918	382.06	212.75	-0.8447	5.36E-153	
cre-miR1172.1	244.36	322.83	0.4017	2.22E-35	
cre-miR1146	222.54	295.10	0.4072	2.90E-33	
cre-miR905*	214.34	531.19	1.3093	0	**
cre-miR1159.1	208.76	378.97	0.8602	1.11E-156	
cre-miR919.1	189.62	114.68	-0.7255	1.56E-59	
cre-miR1153.1*	160.25	140.83	-0.1864	2.48E-05	
cre-miR907	129.22	192.64	0.5761	1.40E-40	
cre-miR908.3	128.85	113.58	-0.1820	2.20E-04	
cre-miR1169	124.65	371.08	1.5739	0	**
cre-miR908.1	113.92	155.65	0.4503	9.79E-22	
cre-miR1151a	93.40	42.69	-1.1295	8.64E-62	**
cre-miR913	71.71	95.46	0.4127	4.55E-12	
cre-miR908.2	67.80	80.57	0.2490	7.94E-05	
cre-miR1151b	62.00	40.90	-0.6000	3.81E-15	
cre-miR1153.2*	61.42	68.42	0.1558	0.02	
cre-miR1150.1	57.65	73.30	0.3465	2.62E-07	
cre-miR1147.1	48.29	333.54	2.7880	0	**

cre-miR909.3	31.76	36.72	0.2092	0.02	
cre-miR1156.2	30.38	10.64	-1.5141	1.77E-32	**
cre-miR910	28.71	430.30	3.9055	0	**
cre-miR1169*	27.77	52.23	0.9112	4.02E-25	
cre-miR1149.2	27.05	18.12	-0.5780	5.19E-07	
cre-miR1150.2	24.65	22.30	-0.1445	0.20	
cre-miR906-5p	23.64	40.63	0.7813	1.16E-15	
cre-miR1144a.2	22.84	46.60	1.0287	2.87E-27	**
cre-miR916	19.94	20.52	0.0413	0.73	
cre-miR1155	18.85	20.52	0.1222	0.32	
cre-miR917	16.17	23.61	0.5460	8.89E-06	
cre-miR915	10.95	7.48	-0.5496	2.30E-03	
cre-miR1158	10.80	4.87	-1.1488	1.15E-08	**
cre-miR906-3p	9.72	7.21	-0.4312	0.02	
cre-miR1159.2	7.90	3.09	-1.3557	2.79E-08	**
cre-miR1157*	6.74	18.60	1.4636	2.19E-19	**
cre-miR909.2	5.73	21.00	1.8742	3.64E-30	**
cre-miR1154*	3.99	2.47	-0.6908	0.02	
cre-miR1145.1	3.70	18.87	2.3515	7.18E-36	**
cre-miR1173	3.55	2.40	-0.5648	0.08	
cre-miR1164	3.12	4.25	0.4485	0.12	
cre-miR1148.2	2.97	10.09	1.7627	3.77E-14	**
cre-miR1160.2	2.54	3.50	0.4637	0.143	
cre-miR1167	1.96	1.65	-0.2493	0.54	
cre-miR911	1.74	13.59	2.9650	1.97E-33	**
cre-miR1142	1.67	1.85	0.1519	0.72	
cre-miR1148.1	1.67	0.75	-1.1435	0.03	*
cre-miR909.1	1.60	2.40	0.5905	0.13	
cre-miR1162*	1.38	0.82	-0.7424	0.16	
cre-miR1144a.1	0.36	2.06	2.5056	2.63E-05	**
cre-miR1165	0.36	15.37	5.4060	2.38E-57	**
cre-miR1163.1	0.07	1.17	4.0081	1.21E-04	**
cre-miR1171	0.14	0		4.85E-30	
cre-miR1143*	0	0.41		1.56E-06	
cre-miR1168.2	0	0.14		4.98E-06	
cre-miR1163.2	0	0.07		1.30E-05	
cre-miR1151b*	0	0.07		1.30E-05	
cre-miR1160.1	0	0.07		1.30E-05	

TAP represent normalized expression level of Known miRNA of *C. reinhardtii* cultivated in nitrogen-replete medium.

TAP-N represent normalized expression level of Known miRNA of *C. reinhardtii* cultivated in

nitrogen-depleted medium.

*Fold-change (log2)>1 or fold-change (log2)< -1, and 0.01<P-value<0.05.

**Fold-change (log2)>1 or fold-change (log2)< -1, and P-value<0.01.
