# Supplementary material for: MicroRNA Expression Profile Analysis of Chlamydomonas reinhardtii during Lipid Accumulation Process under Nitrogen Deprivation Stresses
Source: Bioengineering (Basel). 2021 Dec 27;9(1):6. doi: 10.3390/bioengineering9010006 (PMC8773410; doi:10.3390/bioengineering9010006)

Additional file1. Summary of the common and specific sequences (≥18nt) between TAP-N and TAP libraries

Table S1 Summary of the common and specific sequences .(≥18nt) between TAP-N and TAP libraries

Class	Unique sRNA	%	Total sRNA	%
Total sRNAs	2074319	100.00 %	28362008	100.00 %
Common	220552	10.63 %	25958305	91.52 %
TAP-N specific	1183133	57.04 %	1444335	5.09 %
TAP specific	670634	32.33 %	959368	3.38 %

Figure S1 Ven chart for unique small RNAs

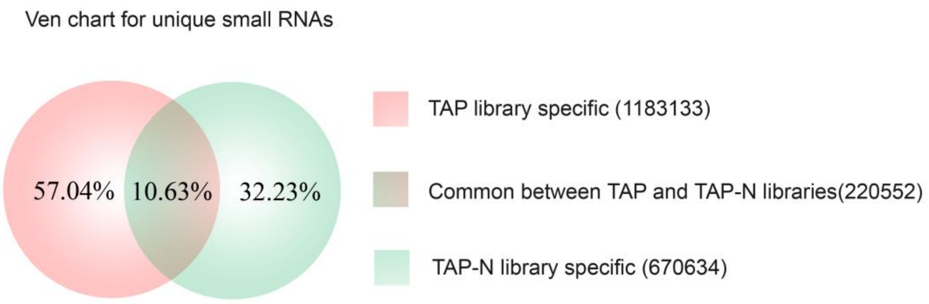

Figure S2 Ven chart for total small RNAs

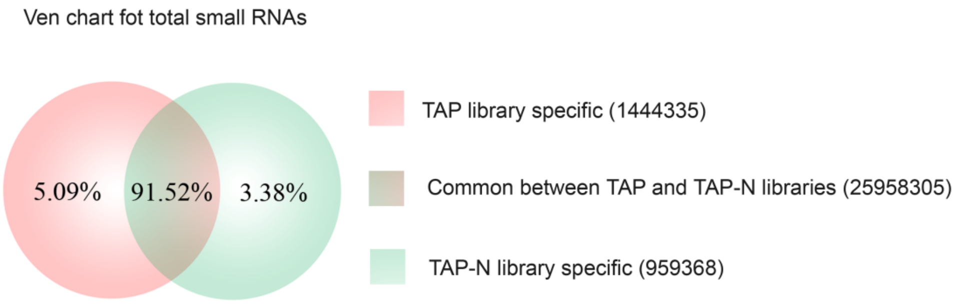

Supplement: Supplementary file 1 [file bioengineering-09-00006-s001.zip › Additional file1.pdf]
